# Trans-styloid, trans-hamate dorsal lunate dislocation: A case report

**DOI:** 10.1016/j.ijscr.2019.07.011

**Published:** 2019-07-19

**Authors:** Anas M. Alyamani, Mohammed F. Alfawzan, Turki S. Alhassan, Obaid M. Almeshal

**Affiliations:** Department of Plastic Surgery, King Abdulaziz Medical City- Riyadh, P.O. Box 22490, Riyadh 11426, Saudi Arabia

**Keywords:** Carpal fracture, Perilunate injury, Dislocation, Lunate, Case report

## Abstract

**Introduction:**

Lunate dislocation is a rare and serious injury, frequently due to high-energy trauma such as a fall from height or a motor vehicle accident. Lunate dislocation is usually in the volar direction; however, dorsal dislocation of the lunate is extremely rare.

**Presentation of case:**

We report a case of 55-year-old male who sustained a high-energy trauma; he was hit by a car while crossing the street. The patient was referred to department of Plastic Surgery for left wrist dorsal dislocation of the lunate and bilateral multiple carpal bone fractures. He was managed by open reduction and internal fixation with scapholunate ligament repair for the left wrist, while the right wrist was treated conservatively.

**Discussion:**

Such cases must be treated urgently to avoid complications such as avascular necrosis of the lunate, median nerve injury, complex regional pain syndrome, and chronic carpal instability.

**Conclusion:**

This case is one of few cases reported in the literature highlighting the rarity of this injury pattern.

## Highlights

• Lunate dislocation is a rare and serious injury, frequently due to high-energy trauma.
• Lunate dislocation is usually in the volar direction; however, dorsal dislocation of the lunate is extremely rare.
• Such cases must be treated urgently to avoid complications such as avascular necrosis of the lunate and median nerve injury.
• This case is one of few cases reported in the literature highlighting the rarity of this injury pattern.

## 1 Introduction

Lunate dislocation is a rare and serious injuries, frequently due to high-energy trauma such as a fall from height or a motor vehicle accident. These injuries are missed clinically and radiographically in up to 25% of cases. In the literature, Herzberg et al. found only one case with dorsal lunate dislocation out of the 166 cases of they reviewed [1].

The importance of identifying these injuries is highlighted by the significant complications produced by missed injuries including avascular necrosis of the lunate, median nerve injury, complex regional pain syndrome, and chronic carpal instability [2], [3], [4].

We hope this report will help to gather more experience for an accurate diagnosis and appropriate prompt management of dorsal lunate dislocation. The work has been reported in line with the SCARE criteria [5].

## 2 Patient information

A 55-year-old male pedestrian was hit by a car while crossing the street. He was brought to the emergency department instantly by an ambulance. The patient was immediately intubated as his Glasgow Coma Scale was 7/15 on presentation. Advanced Trauma Life Support protocol was followed. On the secondary survey, the patient was found to have multiple facial fractures, open fractures on the right forearm and leg, and bilateral wrist injuries. After excluding life-threatening injuries, the patient was referred to department of Plastic Surgery for further evaluation of the bilateral wrist injuries.

## 3 Clinical findings

On examination, both wrists were deformed and edematous; also, he had an open fracture in the right forearm. There was no distal vascular deficit. Peripheral nerves could not be assessed as the patient was sedated and intubated.

## 4 Diagnostic assessment

**Fig. 1:**
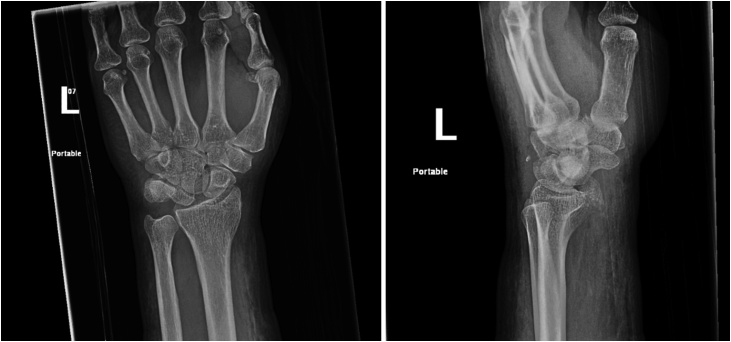
Left wrist X-ray.

Left wrist radiograph showed an intra-articular fracture at the distal end of the radius and a fracture in the distal phalanx of the ring finger. Also, there was a widening of the scapholunate interval. While on the right wrist, distal radius and ulna shaft fractures, and fracture of the triquetrum (Fig. 1).

Computed tomography of the left wrist showed comminuted intraarticular fracture styloid process of the radius, comminuted fracture involving the hamate bone, scapholunate dissociation and advancement of the capitate proximally with consequent dorsal dislocation of the lunate. While on the right wrist, distal radius and ulna shaft fractures, comminuted fracture of the trapezium and triquetrum, and linear fracture of the trapezoid.

## 5 Therapeutic intervention

**Fig. 2:**
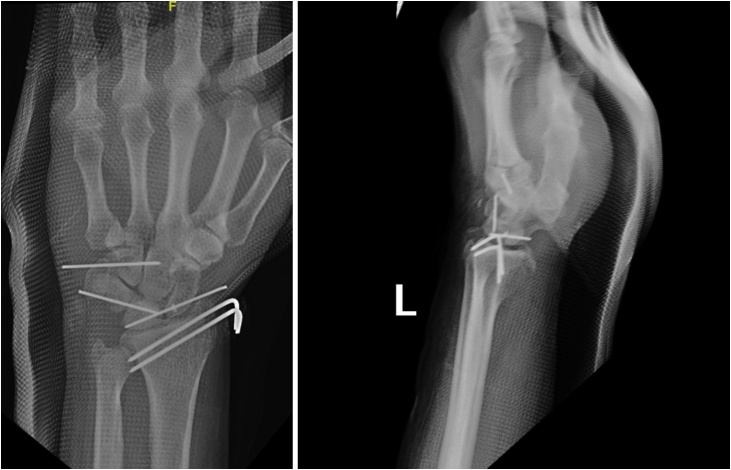
Post-operative X-ray.

After the patient condition stabilized, he underwent open reduction and internal fixation of the left wrist. Under fluoroscopic guidance, two Kirschner (K) wires were introduced to the styloid process of the radius. Combined dorsal and volar approaches were used. A dorsal incision was made, manipulation using a temporary K-wire in the lunate as a joystick. After reduction, two K-wires were introduced to stabilize the scaphoid to the lunate and triquetrum to lunate. Then, the dorsal scapholunate ligament was identified and repaired. The hamate fracture was fixed using a K-wire. A volar incision was made to release the carpal tunnel. After the surgery is completed, below elbow full cast was applied (Fig. 2).

## 6 Discussion

The description of perilunate instability by Mayfield et al. has contributed substantially to our understanding of the perilunate injury and has helped guide operative treatment, which follows the dislocated joints and torn ligaments. In the first stage of this classification, the scapholunate articulation is disrupted. The second stage of progression involves disruption of the capitolunate joint in addition to the scapholunate injury. The third stage of progression is perilunate dislocation where the lunotriquetral joint is also disrupted. The fourth stage of progression is lunate dislocation, where the capitate is still dislocated dorsally from the lunate, but now the radiolunate joint is disrupted and the lunate is dislocated volarly from the lunate fossa on radiographs [6]. Herzberg et al. further classified perilunate instability according to the lateral view on a radiograph to demonstrate the relationships of the capitate, lunate, and radius [1].

Out the 166 cases of perilunate dislocations Herzberg et al. reviewed, he only described one case of dorsal dislocation of the lunate [1]. In the literature, there are a few reports of dorsal lunate dislocatin.

Siddiqi et al. reported a case of dorsal lunate dislocation after a sudden pull by a dog which led to an isolated dorsal lunate dislocation. A single K-wire stabilized the lunate in a Kapandji fashion and protected it from re-dislocation. The authors therefore stated that in patients with trivial trauma a simple K-wire stabilization is a feasible therapeutic option. However, the trauma of our patient is not considered trivial so that we stabilized the lunate by two K-wires [7]. Bjerregaard et al. described a case of radial styloid fracture with associated dorsal dislocation of the lunate in a lorry driver who hit a train who mentioned a combination of hyperflexion, ulnar deviation and pronation of the wrist [8]. Neavin et al. report an unrestrained passenger in a motor vehicle accident who sustained dorsal lunate dislocation, also having fractures to the base of proximal phalanx of the fifth finger and comminuted intra-articular radial styloid fracture [9].

This case was unique in which the extent of the injury was remarkable. Bilateral wrists were injured. On the left side, the lunate was dorsally dislocated, and the patient had comminuted intraarticular fracture styloid process of the radius, comminuted fracture involving the hamate bone, and distal phalanx fracture of the ring finger fracture. While on the right wrist, comminuted fracture of the trapezium and triquetrum, and linear fracture of the trapezoid.

When the treatment is delayed, lunate dislocations are more difficult to reduce, and outcomes may be jeopardized, leading to nonunion, carpal arthritis, and collapse [10]. Therefore, this case was managed urgently after ruling out life-threatening conditions.

The management of lunate dislocations and fracture dislocations remains controversial. Internal fixation such as K-wire fixation, K-wires plus external fixation, minimally invasive procedures or open surgery are discussed in the literature [11]. Furthermore, a widened scapholunate gap as seen in our patient indicates an injury to the scapholunate ligament which can be addressed by open repair.

In summary, we have described a rare case of trans-styloid trans-hamate lunate dorsal dislocation after sustaining a high-energy trauma. Treatment with open reduction and internal fixation necessitated volar and dorsal approaches as well as repair of scapholunate ligament.

## Funding

There is no financial support nor sponsorship.

## Ethical approval

This study was approved by the Ethics Committee at the national guard health affairs.

## Consent

Written informed consent was obtained from the patient for publication of this case report and accompanying images.

## Author contribution

Anas Alyamani: Writing paper, literature review and data collection.

Turki Alhassan: Writing paper, literature review and data collection.

Mohammed Alfawzan paper reviewer.

Obaid Almeshal: Main Surgeon, outpatient clinic consultation and data collection.

## Registration of research studies

Not applicable.

## Guarantor

Anas Alyamani.

## Declaration of Competing Interest

The authors report no conflicts of interest. The authors alone are responsible for the content and writing of the paper.
